# Sulforaphane Exerts Beneficial Immunomodulatory Effects on Liver Tissue *via* a Nrf2 Pathway-Related Mechanism in a Murine Model of Hemorrhagic Shock and Resuscitation

**DOI:** 10.3389/fimmu.2022.822895

**Published:** 2022-02-10

**Authors:** Weiqiang Liang, Johannes Greven, Kang Qin, Athanassios Fragoulis, Klemens Horst, Felix Bläsius, Christoph Wruck, Thomas Pufe, Philipp Kobbe, Frank Hildebrand, Philipp Lichte

**Affiliations:** ^1^ Department of Orthopaedics, Trauma and Reconstructive Surgery, University Hospital Rheinisch-Westfälische Technische Hochschule (RWTH) Aachen, Aachen, Germany; ^2^ Department of Bone and Joint Surgery, The First Affiliated Hospital of Shandong First Medical University, Jinan, China; ^3^ Department of Anatomy and Cell Biology, Rheinisch-Westfälische Technische Hochschule (RWTH) Aachen University, Aachen, Germany

**Keywords:** hemorrhagic shock/resuscitation, liver, ischemia-reperfusion injury, cytokines, Kupffer Cells, sulforaphane, Nrf2, KEAP1

## Abstract

Our research explores the immunomodulatory effects of sulforaphane (SFN), a well-known nuclear factor erythroid 2-related factor 2 (Nrf2) pathway agonist, on the sterile inflammation of and ischemia-reperfusion injuries to the liver after hemorrhagic shock (HS) followed by resuscitation (R). Male C57/BL6 wild-type and transgenic ARE-*luc* mice were exposed to mean arterial pressure-controlled HS. Fluid resuscitation was performed after 90 min of HS, and SFN was administrated intraperitoneally after that. The animals were sacrificed at 6 h, 24 h, and 72 h after resuscitation, and their livers were extracted to perform H&E staining and myeloperoxidase (MPO) activity analysis. The Kupffer cells were isolated for cytokines profile measurements and Nrf2 immunofluorescence staining. Further, the ARE-*luc* mice were used to assess hepatic Nrf2 activity *in vivo*. We identified that SFN-activated Kupffer cells’ Nrf2 pathway and modulated its cytokines expression, including TNF-α, MCP-1, KC/CXCL1, IL-6, and IL-10. Furthermore, SFN mitigated liver ischemia-reperfusion injury, as evidenced by the downregulation of the Suzuki score and the enhanced hepatic Nrf2 activity. The *in vivo* SFN treatment decreased neutrophils infiltration, as shown by the decreased MPO levels. Our study shows that SFN can decrease HS/R-induced hepatic ischemia-reperfusion injury and modulate the activity of Kupffer cells *via* an Nrf2-dependent pathway.

## Introduction

Hemorrhagic shock (HS) remains one of the leading causes of death in traumatized patients (1) and negatively affects multiple organs of survivors by causing hypoxia, cell damage, and organ failure. This process is accompanied by a sterile inflammatory response that includes an overwhelming oxidative stress reaction (2). For the liver, as a central organ in homeostasis, a deficiency in blood supply may disrupt the hepatic cellular metabolism, leading to liver dysfunction and even failure, which is a fatal complication that leads to increased mortality rate. Hepatic Kupffer cells are well known to play a central role in these processes (3–5). As local resident macrophages, Kupffer cells serve as a major source of proinflammatory cytokines and reactive oxygen species (ROS), which are liberated during the early stages of HS (6).

The nuclear factor erythroid 2-related factor 2 (Nrf2) has been proven to exert hepatoprotective effects in case of toxin induced hepatitis. It belongs to the family of basic region leucine zipper (bZIP) transcription factors. In homeostatic conditions, Nrf2 is sequestered in the cytosol *via* the physical attachment of the N-terminal domain to the Kelch-like ECH-associated protein 1 (Keap1), which, in turn, causes the inhibition of Nrf2 activity (7). Upon the increased occurrence of oxidants, Nrf2 dissociates from Keap1 and translocate into the nucleus, interacts with the antioxidant response element (ARE), which is a DNA motif located in the upstream promoter regions, and, finally, mediates the expression of several antioxidant enzymes (8). Due to the aforementioned aspects, Nrf2 has been considered as a target for new therapeutic approaches in the treatment of several liver diseases (9).

Sulforaphane (SFN), a natural isothiocyanate, is a well-known Nrf2 pathway agonist (10). It has been reported that SFN activates Nrf2 activity in peritoneal macrophages and exerts anti-inflammatory effects *via* the inhabitation of TNF-α, IL-1β, COX-2, and iNOS expression in a murine model (11). In our previous publication, we reported the beneficial effects of *in vivo* SFN administration on lung ischemia-reperfusion damage and the systemic inflammatory response in an isolated murine HS/R model (12). It was indicated that SFN exerts anti-inflammatory effects by down-regulating multiple pro-inflammatory alveolar macrophages (AMs) generated cytokines, during which process accompanies AMs’ Nrf2 pathway activation. 

In this study, we investigated the effects of SFN on HS/R-induced liver damage because compared to other organs, the liver is actually well-protected from general ischemic injury due to its dual blood flow system. This system assures a rescue circulation since the blood received by the liver is shared between the portal vein and the hepatic artery (13, 14). Additionally, the highly permeable sinusoids of the liver allow increased oxygen diffusion to hepatocytes, increasing oxygen extraction to levels approaching 90% (15). However, the severe hypovolemia that occurs in the course of hemorrhagic shock can overcome even these robust supportive mechanisms and consequently damage the liver. This is of importance because the liver is the central organ of metabolism, and therefore a dysfunction of the hepatocytes has further serious consequences for the entire body. Because Nrf2 activity has been shown to be beneficial in a variety of liver injury models (9, 16–18) not only because of its cytoprotective and antioxidant properties but also because of its marked influence on metabolic pathways (19–21), the investigation of the effects of SFN-induced Nrf2 activity in our HS/R model was of great interest.

Our present study will elucidate the possible effects of SFN on liver inflammation after isolated HS/R and Nrf2-related mechanisms.

## Materials and Methods

With regard to the 3R-criteria by Russell and Burch (22), the presented results are taken from a larger project of which partial data have been published already (12).

### Animal Care and Experimental Design

Our research was approved by the North Rhine-Westphalia Animal Welfare Committee (AZ 84-02.04.2017.A269). We selected male C57/BL6 wild-type (WT) mice (Charles Rivers Laboratories, Germany) and transgenic ARE-*luc* mice (Cgene, Oslo, Norway) that were 8–10 weeks old and weighed 20–30 g.

All the animals were kept for more than one week to prevent stressive factors from interfering with the stress/inflammation-related measurements. In total, 78 WT mice were divided into five groups (control, sham, HS/R, sham + SFN, and HS/R + SFN) and tested at three points of time (6 h, 24 h, and 72 h) after HS/R (**Table 1**). The number of animals was set by statistical power analysis *via* setting the confidence level at 95%, which ensured 6 animals per group was rational and statistically significant (http://powerandsamplesize.com/Calculators/Compare-k-Means/1-Way-ANOVA-Pairwise-1-Sided; https://clincalc.com/stats/samplesize.aspx). The control animals were sacrificed immediately to obtain samples, and the experimental mice were sacrificed at 6 h, 24 h, and 72 h post resuscitation. The sham animals were sacrificed at 6 h, 24 h, and 72 h post operation. Thoracotomy (with anesthesia using pentobarbital and desflurane) was performed, and the mice were exsanguinated *via* cardiac puncture. Liver tissues were collected from WT mice for myeloperoxidase (MPO) testing, hematoxylin and eosin (H&E) staining, and Suzuki score calculation. Kupffer cells were isolated and cultured for cytokine measurement. In total, 24 ARE-*luc* mice were allocated to each group (six per group) for testing the hepatic Nrf2 activity at 0 h (before surgical procedure), 6 h, and 24 h post HS/R and sham surgery. Each of ARE-*luc* mice was tested 3 times before sacrificed.

**Table 1:** Groups distribution of WT and ARE-luc mice.

Termination post-intervention	Genotype	Control	Sham + vehicle	Sham + SFN	HS/R + vehicle	HS/R + SFN
0 h	C57/BL6 (WT)	6				
6 h	C57/BL6 (WT)		6	6	6	6
24 h	C57/BL6 (WT)		6	6	6	6
72 h	C57/BL6 (WT)		6	6	6	6
24 h	C57/BL6 (ARE-luc)		6	6	6	6

### Anesthesia and Induction of HS/R

Analgesia was performed *via* preoperative (30 min) buprenorphine (Temgesic^®^, 0.05–0.1 mg/kg/body weight, s.c.) injection. The inhalation anesthesia was initiated with Desflurane (6%, MAC 50) in an induction box, and the anesthesia was maintained by inhalation anesthesia (1.5%, MAC 50). The mice were placed on a warming pad in a supine position. A unilateral incision of about 1 cm was made to expose the femoral artery of the left femur, which was intubated with a sterile PE-10 polyethylene catheter (Braintree Scientific Inc., USA). Heparin (Baxter Healthcare Corporation, Deerfield, IL) (0.5 IU/g BW each) was used as an anticoagulant in the syringe during blood withdrawal and 90-min HS. The volume of blood withdrawal was shown in supplementary material (**Supplementary Table S1**). The catheter was connected to a digital blood pressure monitor (Digi-Med ^®^, Louisville, KY, USA), and the HS condition (MAP of 35–45 mmHg) was maintained for 90 min. The withdrawn blood was reinfused *via* infusion pump along with a 0.9% saline solution (two times the volume of the drawn blood) to perform resuscitation (0.2 ml/min). Protamine (0.5 IU/g BW each) was injected immediately after resuscitation *via* the catheter to counteract the heparin’s effects. Finally, the PE catheter was removed, and the artery was ligated. Additional buprenorphine (0.05–0.1 mg/kg) was administered every 6–8 h post-surgery. The sham mice underwent the same surgical procedure without blood withdrawal.

### SFN Administration

SFN (CAS registry number:142825-10-3) (50 mg/kg) was injected intraperitoneally after resuscitation in the Sham + SFN and HS/R + SFN groups. The solvent for SFN (0.9% saline) was used as the vehicle. The first SFN injection was 1.5h after hemorrhagic shock and resuscitation. In 6h and 24h groups, SFN was administrated once at 1.5h, while in 72h groups, SFN was injected 3 times at 1.5h, 24h and 48h.

### Bioluminescence Imaging (BLI) of Hepatic Nrf2 Activity

The hepatic Nrf2 activity *in vivo* was evaluated with the male ARE-*luc* transgenic mice. The transcription of the luciferase gene is controlled by ARE, and the bioluminescence directly correlates with the Nrf2 activity. Measurements were performed three times for each mouse: Control measurements were performed before surgery for the determination of the baseline levels. Further measurements were performed at 6 h and 24 h after HS/R or sham surgery. A 200μl D-luciferin solution (containing 4 mg of D-luciferin; Synchem, Switzerland) was injected intraperitoneally under Desflurane inhalation anesthesia 10 min before testing, as described by Fet et al. (23). The front upper abdominal hepatic area was selected as the region of interest (ROI), and the images were recorded using a Xenogen Ivis^®^ Lumina System. The Living Image Software 2.5 (Caliper Life Sciences, California) was used to quantify the photons emitted. The signal intensity was quantified as the sum of all photons counts per second within the ROI with reference to the background luminescence of the lower limbs. The results were presented as average radiance (photons/second/square centimeter/steradian: p/s/cm^2^/sr).

### Liver H&E Staining and Ischemia-Reperfusion Injury Evaluation

The right lateral lobes of the livers of the WT mice were excised under sterile conditions immediately after euthanasia and embedded in paraffin before H&E staining were performing. The liver tissues were sliced into 5–7 μm thick pieces using a microtome (Leica Biosystems, Heidelberger, Germany). Histological observation was performed using an optical microscope (Carl Zeiss, Jena, Germany) with a magnification of 200 times. Two independent researchers (WL and JG) assessed the acute liver injury scores according to the Suzuki standards (24).

### MPO Testing

The accumulation of polymorphonuclear leukocytes in the livers was assessed by MPO activity. The left medial lobe of the livers from the WT mice were snap frozen in liquid nitrogen and stored at -80°C before MPO testing. The radioimmunoprecipitation (RIPA) assay buffer was used to prepare tissue homogenates by tissue lysis. An MPO-enzyme-linked immunosorbent assay (ELISA) kit (Hycultec GmbH, Beutelsbach, Germany) was used according to the manufacturer’s instructions.

### Isolation and Immunostaining of Kupffer Cells’ Nuclear Nrf2 Translocation and Cyto-Plastic Keap1

The Kupffer cells were isolated with collagenase digestion and Percoll gradient centrifugation as previously described (25). The remaining lobes of the livers from the WT mice were perfused by HBSS (Thermo Fisher Scientific, USA) *via* the portal vein before removal. Thereafter, the liver tissues were incubated in a collagenase IV solution (Worthington, Lakewood) for 15 min at 37°C. Some of the isolated Kupffer cells was transferred to a six-well plate with a cell count of 5 × 10^5^ per well and cultured in an incubator (37°C, 95% humidity, and 5% CO_2_) for 24 h before the supernatant culture medium was stored at -80°C for ELISA testing.

The other two aliquots of Kupffer cells were immediately fixed with 4% paraformaldehyde Phosphate buffered saline (PBS) solution (pH = 7.4) at room temperature for immunofluorescence staining. The fixed Kupffer cells were then permeabilized with PBS containing 0.25% Triton X-100. After blocking with PBS containing 10% goat serum, the cells were incubated with anti-Nrf2 antibody (Abcam 137550, England, 1:500) and anti-Keap1 antibody (Abcam 139729, London, England, 1:200) separately overnight. Then, the Kupffer cells were stained with goat anti-rabbit IgG H&L (DyLight1 488, Abcam 96895, 1:500) pre-adsorbed secondary antibodies for 1 h at room temperature. The nuclei were counter-stained with 4′,6-diamidino-2-phenylindole (DAPI). The subcellular localization of Nrf2 and cytoplastic Keap1 was observed in 20 microscopic fields (5–10 cells/field) by two independent researchers (WL and JG) with confocal fluorescence microscopy (Zeiss Axiovert 200M, Germany). Quantitative analyses of the mean density of fluorescence in the nuclei and cytoplasm were performed with the AxioVision Rel. 4.8 software (Carl Zeiss MicroImaging, LLC).

### Assessment of Cytokines in Kupffer Cells’ Culture Supernatant

The Kupffer cells’ culture supernatant was tested for TNF-α, IL-6, human macrophage chemoattractant protein-1 (MCP-1), keratinocyte (KC), granulocyte macro-phage colony-stimulating factor (GM-CSF), and IL-10. These cytokine and chemokine levels were evaluated using mouse premixed multi-analyte kits (R&D System Inc., Minneapolis, MN, USA) according to the manufacturer’s manuals.

### Serum ALT and AST Levels

Serum ALT and AST levels were tested with auto-biochemical analyzer by a well-trained technician and were expressed as U/L.

### Statistical Analysis

The Shapiro–Wilk test was used to determine the data distribution. Normally distributed data were expressed as mean ± SD. The interquartile range (IQR) was used if the data were non-normally distributed. Differences between the groups were determined by one-way ANOVA followed by Tukey’s *post hoc* test for normally distributed data, and the non-normally distributed data were assessed using the Kruskal-Wallis test followed by Dunn’s *post hoc* test. The statistical significance threshold for all analyses was set at p < 0.05. The statistical calculations were performed using SPSS (version 24.0.0; IBM, Armonk, NY, USA).

## Results

### SFN’s Effects on Hepatic Nrf2-ARE Activity

**Figure 1:**
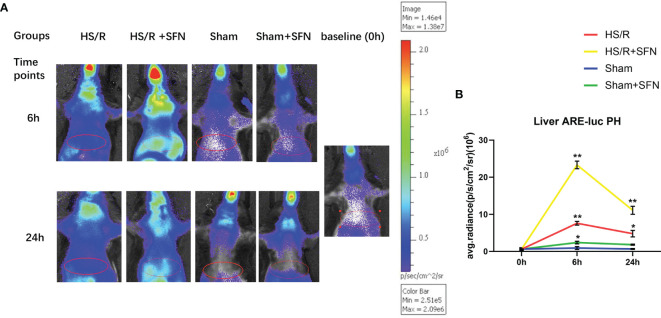
Bioluminescence emission of the liver region of ARE-luc mice (n=6 in each group). The ARE-luc transgenic mice underwent an HS/R or sham operative procedure. The luciferase activity *in vivo* was measured *via* BLI after the intraperitoneal injection of luciferin preoperatively (0 h) and post operatively (6 h and 24 h). The red ellipses mark the selected ROIs used to evaluate signal intensity. **(A)** The representative animals for each group and time point. High signal intensities were also observed around the neck, chest, and the nasal and oral cavity. **(B)** The *in vivo* hepatic luciferase activity was recorded as the average radiance in photons per second per cm^2^ per steradian (p/s/cm^2^/sr), which represents the mean values for six animals and the standard deviation (*p < 0.05, **p < 0.01; One-way ANOVA followed by Tukey’s *post hoc* test).

The BLI of the transgenic ARE-*luc* mice demonstrated that the average luminescence after HS/R was significantly increased from the baseline level of 1.2 ± 0.3×10^6^ p/s/cm^2^/sr to a peak level of 7.2 ± 0.9 × 10^6^ p/s/cm^2^/sr at 6 h post HS/R (p < 0.01). The SFN-treated HS/R mice showed enhanced hepatic Nrf2-ARE activity compared to the vehicle-treated mice, with a peak level being observed at 6 h after resuscitation (23.9 ± 3.1 × 10^6^ p/s/cm^2^/sr) (p < 0.01). The hepatic region of the sham animals also showed increased Nrf2-ARE activity after SFN treatment. However, the peak level of luminescence was much lower than that of the HS/R animals (3.1 ± 0.8 ×10^6^ p/s/cm^2^/sr) (**Figure 1**).

### Effects of SFN on Nrf2 Translocation Into Kupffer Cells’ Nuclei

**Figure 2:**
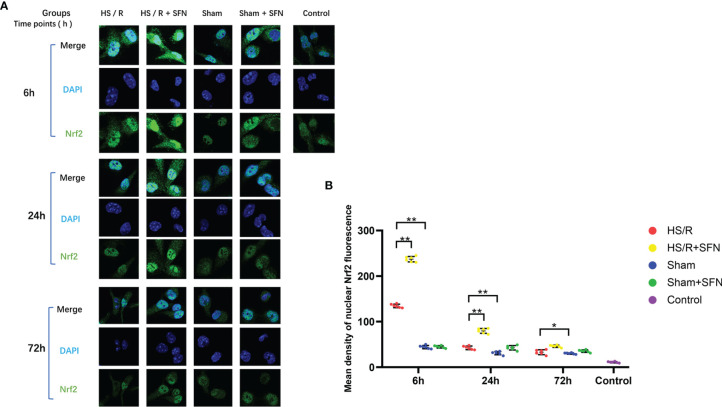
Immunofluorescence analysis of Nrf2 nuclear translocation (n=6 in each group). **(A)** Kupffer cells were isolated from the WT mice that underwent the different experimental procedures and were fixed immediately. Nrf2 was probed with a primary anti-Nrf2 antibody and visualized with a goat anti-Rabbit DyLightH 488-conjugated secondary antibody. The nuclei were stained with DAPI. The images were taken using confocal fluorescence microscopy. **(B)** The quantitative analyses of the mean intensity of fluorescence (λ = 488nm) in the nuclei were performed with the AxioVision Rel. 4.8 software (Carl Zeiss Micro Imaging, LLC) (*p < 0.05, **p < 0.01; One-way ANOVA followed by Tukey’s *post hoc* test).

The average fluorescence intensity of Nrf2 in the Kupffer cells’ nuclei of the HS/R mice was significantly higher than that of the sham animals at 6 h (p < 0.01) and 24 h (p < 0.05) after HS/R. The highest Nrf2 nuclear accumulation for the HS/R animals was observed at 6 h post resuscitation. The SFN treatment significantly elevated the average fluorescence intensity of nuclear Nrf2 at 6 h (p < 0.01), 24 h (p < 0.01), and 72 h (p < 0.05). The Kupffer cells’ average nuclear Nrf2 fluorescence intensity for the sham mice was not significantly affected by SFN treatment (**Figure 2**). Negative control of immunofluorescence is shown in supplementary material (**Supplementary Figure S1**).

### Immunofluorescence Imaging of Kupffer Cells’ Cytoplasmic Keap1

**Figure 3:**
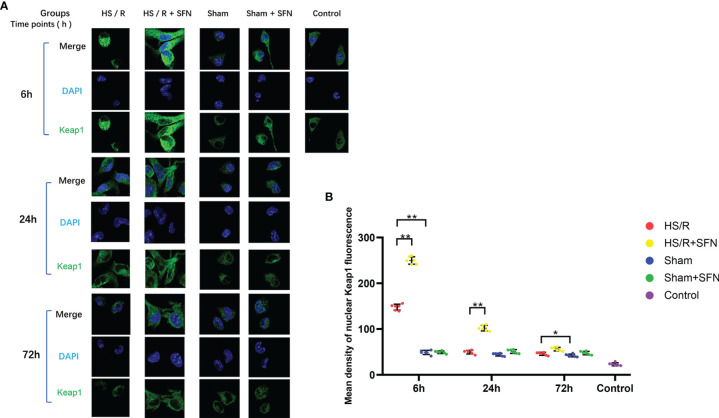
Immunofluorescence analysis of cytoplasmic Keap1 (n=6 in each group). **(A)** Kupffer cells were isolated from the WT mice of different groups and fixed immediately. Keap1 was targeted with a primary anti-Keap1 antibody and visualized with a goat anti-Rabbit DyLightH 488-conjugated secondary antibody. The nuclei were stained with DAPI, and the images were taken using confocal fluorescence microscopy. **(B)** The quantitative analyses of the mean density of fluorescence in the nuclei were performed with the AxioVision Rel. 4.8 software (Carl Zeiss Micro Imaging, LLC) (*p < 0.05, **p < 0.01; One-way ANOVA followed by Tukey’s *post hoc* test).

The average fluorescence density of the Kupffer cells’ cytoplasmic Keap1 of the HS/R mice was significantly higher than that of the sham animals at 6 h (p < 0.01) post fluid resuscitation. SFN treatment after HS/R significantly up-regulated the average fluorescence density of cytoplasmic Keap1 at 6 h (p < 0.01), 24 h (p < 0.01), and 72 h (p < 0.05). The average fluorescence density of the Kupffer cells’ cytoplasmic Keap1 of the sham mice was not modulated by SFN treatment (**Figure 3**).

### SFN’s Effects on the Cytokine Secretion of Kupffer Cells

**Figure 4:**
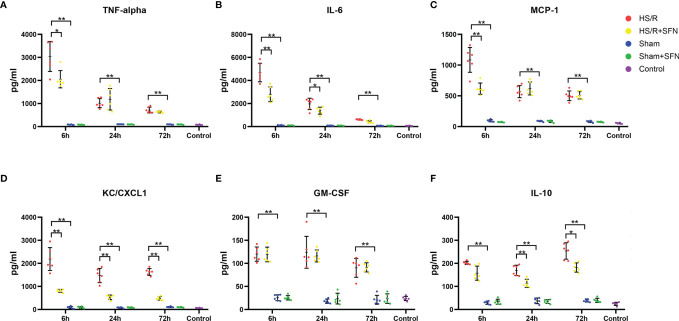
Cytokines secreted by Kupffer cells (n=6 in each group). The TNF-α **(A)**, IL-6 **(B)**, MCP-1**(C)**, KC/CXCL1 **(D)**, GM-CSF **(E)**, and IL-10 **(F)** levels in C57/BL6 WT mouse plasma following HS/R or sham surgery with the HS/R + SFN group or sham + SFN group or without the administration of SFN (HS/R groups or sham groups). The results are expressed in pg/mL as mean ± SD. (*p < 0.05, **p < 0.01; the data of 6h group in graph **(A)** were analyzed by Kruskal-Wallis test followed by Dunn’s *post hoc* test, and others were obtained with use of one-way ANOVA followed by Tukey’s *post hoc* test).

HS/R led to significant enhancement in the Kupffer cell secretion of TNF-α, IL-6, MCP-1, KC, GM-CSF and IL-10 at three time points (6 h, 24 h, and 72 h) as compared to the sham group. The Kupffer cells of the mice that underwent *in vivo* SFN treatment after HS/R showed a significantly decreased expression of TNF-α (6 h), IL-6 (6 h and 24 h), MCP-1 (6 h), KC (6 h, 24 h, and 72 h), and IL-10 (24 h and 72h) compared to the vehicle-treated groups, while GM-CSF secretion was not significantly altered (**Figure 4**). The cytokine secretion of the sham mice was not influenced by SFN treatment.

### SFN’s Effects on Hepatic MPO Activity

**Figure 5:**
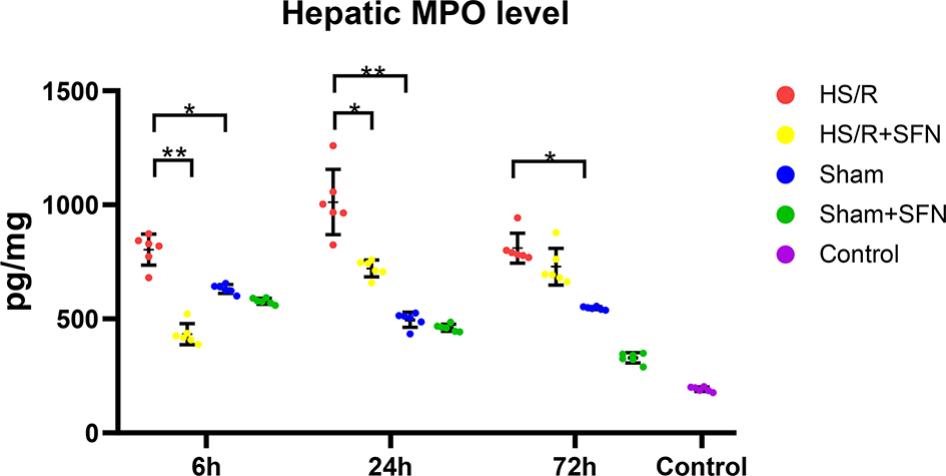
Hepatic MPO activity (n=6 in each group). C57/BL6 WT mice following HS/R or sham surgery with HS/R + SFN or sham + SFN or without the administration of SFN (HS/R groups or sham groups). The results are expressed as pg/ml, data = mean ± SD (*p < 0.05, **p < 0.01; One-way ANOVA followed by Tukey’s *post hoc* test).

The HS/R induced significantly elevated MPO activity at 6 h (p < 0.05), 24 h (p < 0.01), and 72 h (p < 0.05) post HS/R as compared to the sham animals. The *in vivo* SFN treatment was associated with a significant reduction in hepatic MPO activity as compared to the vehicle-treated animals at 6 h (p < 0.01) and 24 h (p < 0.05) post HS/R. The application of SFN had no significant effect on the MPO activity of the sham mice in our experiment (**Figure 5**).

### Histological Analysis and Serum ALT and AST Level

**Figure 6:**
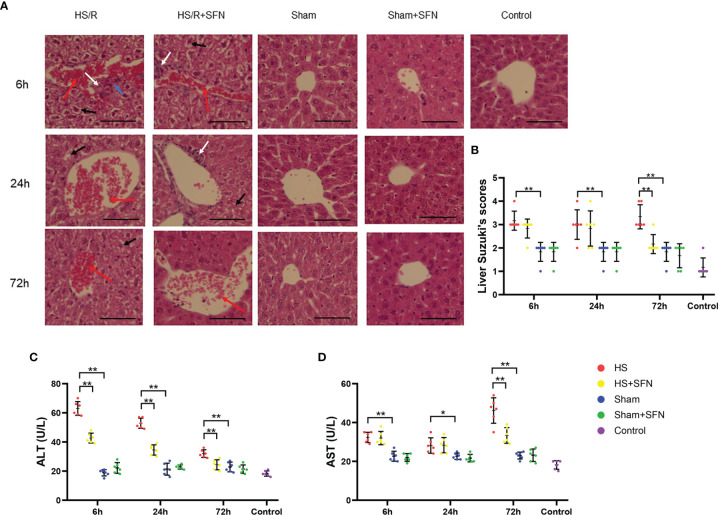
**(A)** H&E staining liver (n=6 in each group; 200× magnification). H&E staining was performed to visualize cells and determine pathophysiological changes in them (blue arrows: hepatocyte necrosis; black arrows: hepatocyte vacuolation and steatosis; red arrows: portal vein congestion; white arrows: neutrophil infiltration). Scale bar = 50μm. **(B)** The liver injury score (according to the Suzuki standard). The liver injury score was calculated according to the scoring system that ranged from 0 to 5, as mentioned above. Every field of the sample was evaluated by two independent observers (*p < 0.05, **p < 0.01; Kruskal-Wallis test followed by Dunn’s *post hoc* test). ALT **(C)** and AST **(D)** levels in C57/BL6 WT mouse plasma were expressed in U/L as mean ± SD. (n=6 in each group; *p < 0.05, **p < 0.01; One-way ANOVA followed by Tukey’s *post hoc* test).

Abnormal histological changes in the sham animals were minimal, with typical liver tissue sections that showed clear liver lobule structures. The HS/R animals showed varying degrees of inflammatory cell infiltration, loss of liver lobule tissue structure, including necrosis, steatosis, portal vein congestion, and interstitial edema, or haemorrhage. Overall, our research demonstrated a significantly higher acute liver Suzuki injury score in the HS/R animals than the sham animals (6 h, 24 h, and 72 h; p < 0.01). *In vivo* SFN administration is associated with a significant decrease in the Suzuki injury score at 72 h post-HS/R (p < 0.01) as compared to the HS/R animals that were treated with the vehicle (**Figures 6A, B**). The HS/R induced significantly elevated serum ALT level at 6 h, 24 h and 72 h (all p < 0.01) as compared to the sham groups. SFN treatment was associated with a significant reduction of serum ALT level as compared to the vehicle-treated animals at 6 h, 24 h and 72h (all p < 0.01) post HS/R. Serum AST significantly elevated at 6 h (p < 0.01), 24 h (p < 0.05) and 72 h (p < 0.01) post HS/R. SFN treatment down-regulated serum AST level as compared to the vehicle-treated animals at 72h (p < 0.01) post HS/R (**Figures 6C, D**).

## Discussion

HS is associated with an overwhelming formation of ROS, which promotes the liberation of multiple cytokines by hepatic localized Kupffer cells (26). Nrf2 is widely recognized as a key protective pathway against oxidative stress and hepatic inflammation (27). However, the potential beneficial effects of SFN, one of the most well-known Nrf2-ARE pathway agonists, for isolated HS/R-induced liver ischemia-reperfusion injury and sterile inflammation have not yet been sufficiently explored.

The main results of our study can be summarized as follows:

1. *In vivo* SFN administration significantly promotes hepatic Nrf2-ARE activity. After HS/R it contributes to both, Kupffer cells’ cytoplasmic Nrf2-Keap1 disintegration and, in turn, Nrf2 translocation into their nuclei.
2. *In vivo* SFN treatment downregulates the isolated HS/R-induced proinflammatory cytokines, including TNF-α, MCP-1, IL-6, and KC as well as the anti-inflammatory cytokine IL-10 expressed by Kupffer cells.
3. *In vivo* SFN administration alleviates liver ischemia-reperfusion injury post HS/R and decreases MPO level.

Kudoh et al. (28) reported that the depletion of Nrf2 in mice aggravated hepatic inflammation and oxidative stress after ischemia and reperfusion (I/R), while the activation of Nrf2 significantly prevented hepatic damage. Accordingly, the results presented in the current study with increased Nrf2 activity after HS/R might be considered a compensative protective mechanism. The Kupffer cells’ Nrf2 was most significantly translocated into the nucleus at 6 h post HS/R, while it showed reduced nucleus Nrf2 density later. Accordingly, the mean density of the cytoplastic Keap1 fluorescence showed a similar trend. This finding could be based on higher levels of possibly induced oxidative stress which might occur immediately after HS/R. Nevertheless, the observed natural Nrf2 activation seems to be insufficient to protect the liver from I/R damage. In fact, SFN promotes the expression of endogenous antioxidants, which are able to reduce oxidative stress. In this context, SFN has been shown to reduce oxidative stress and inflammation by activating Nrf2. Alam et al. and Fragoulis et al. (9, 29) demonstrated that SFN treatment modifies the Keap1 cysteine residues and promotes its disintegration with Nrf2, which, in turn, exerts potent Nrf2 pathway activating effects. The protective function of Nrf2 with regard to the oxidative stress on liver injuries is well described (30). In line with the current literature, we observed that *in vivo* SFN treatment promoted Kupffer cells’ Nrf2 disintegration with Keap1. This was accompanied by an activated Kupffer cell Nrf2 pathway even beyond the very early phase (6 h after HS/R) and a decreased secretion of multiple proinflammatory cytokines, which may help attenuate I/R damage in the liver.

The local inflammatory status of the liver is profoundly affected by Kupffer cells. Previous studies reported that Nrf2 suppresses the macrophage-based inflammatory response by blocking proinflammatory cytokine transcription and acts as an upstream regulator of cytokine production (31). In this context, Kobayashi et al. (32) also found that Nrf2 interferes with the transcriptional upregulation of IL-6 *via* binding to the proximity of the gene and inhibits RNA Pol II recruitment in macrophages. This leads to a decreased secretion of IL-6, which is in line with the findings of this study.

It is well known that TNF-α activates cell-death pathways (33), and, therefore, the decrease in Kupffer cells’ TNF-α expression of SFN-treated mice at 6 h post HS/R might be beneficial for alleviating hepatic necrosis. The Kupffer cells-produced IL-10 reached peak levels at 72 h post HS/R, while the proinflammatory cytokines’ peak levels all occurred at 6 h. This may be interpreted as a classic counter balance mechanism, which was also demonstrated in polytrauma patients (SIRS/CARS) (34). Unexpectedly, we found not only pro- but also anti-inflammatory was down regulated by SFN. However, the ration of IL-6/IL-10 of HS/R group in 6h is 23.2, which is 17.8 of HS/R+SFN group. It showed that the degree of IL-6 decrease was much higher than that of IL-10 and indicated SFN exhibited anti-inflammatory properties for liver I/R injury. SFN did not show direct effects on IL-10 production at 6 h after HS/R but demonstrated a significant down-regulating effect on IL-10 at 24 h and 72 h. This could be caused directly by SFN itself or, more likely, could be driven by the lower proinflammatory trigger due to the decreased levels of proinflammatory cytokines. GM-CSF promotes inflammation in various inflammatory diseases and is generally assumed to be an efficient activator of M1 macrophage polarization (35). KC is a classical marker that indicates a potential transition from the alternative M2 to a more M1 proinflammatory phenotype (36). By contrast, IL-10 promotes reduced M1 macrophage activation and increased M2 macrophage activation (37). Thus, we can conclude that it is possible that sterile inflammation following isolated HS/R induces more Kupffer cells to polarize to the proinflammatory M1 phenotype and leads to the generation of more proinflammatory cytokines. Furthermore, Kupffer cell-produced GM-CSF may result in a positive feedback loop that augments M1 polarization (38). Nakasone et al. (31) found SFN to exert effects on the phenotypic differentiation of macrophages at non-cytotoxic concentration. SFN has the effect of shifting monocytes to specific M2-type macrophages or convert M1-polarized cells into M2 macrophages *via* a MAPK-related mechanism, which maybe another underlying explanation for our findings. Our findings indicate that SFN may yield the beneficial effects of promoting Kupffer cells’ Nrf2 activation and modulating the expression of various cytokines that can potentially alleviate isolated HS/R-induced hepatic I/R injuries.

Oxidative stress and elevated inflammatory cytokines not only lead to harmful systemic effects but also potentially damage local organ tissue. Regarding the liver, inflammatory changes result in severe centrilobular congestion and hepatic dysfunction that are characterized by necrosis and vacuolization (39). Serum AST and ALT levels are the most useful indicators of liver cell injury (40). We observed significantly increase of ALT and AST post HS/R accompanying liver tissue injury as compared with sham animals.

In our study, SFN treatment for HS/R animals also led to significantly elevated Nrf2 activity of the liver tissues as compared to untreated animals. This was accompanied by significantly reduced Suzuki scores, MPO, ALT and AST levels post resuscitation. The alleviation of the liver injury may be closely related to possible lower levels of the oxidative stress which is indirectly indicated the determined higher Nrf2-ARE pathway activation, nevertheless not indicating the oxidative stress levels directly. Although MPO produced by both Kupffer cells and neutrophils, as document by literature, MPO is mainly derived from neutrophil infiltration following liver tissue injury (41). In our present research, we found SFN exerts protective effect and attenuate necrosis, which may help reduce neutrophil infiltration and MPO level. The peak MPO level in the liver tissues at 6 h after HS/R may be also closely related to the elevated Kupffer cells’ production of chemokines, especially MCP-1 and KC. We found SFN down-regulated Kupffer cells produced MCP-1 and KC, which may help reduce MPO level. Overwhelming oxidative stress promotes higher neutrophil infiltration in liver tissues as well. In addition, there is evidence showing that vascular endothelial function may be disrupted by neutrophil MPO *via* oxidative stress (42), which may be related to the severe portal venous congestion that we found in the HS/R mice. SFN administration decreased the liver MPO level at 6 h and 24 h after HS/R, which may also attribute to the Nrf2 pathway activation.

For the first time, SFN as a well-known Nrf2 pathway agonist, was applied to a rodent isolated HS/R model to observe its effects on Kupffer cells participating in hepatic sterile inflammation. Nevertheless, our study obtained positive results for SFN treatment of HS/R it underlies minor limitations. First, all operations are performed under general anesthesia in animals, which may cause regulation of cell damage. To avoid an unwanted bias, we included suitable sham and control groups in the experimental setup to be able to calculate and subtract possible shifts of the baseline from the data of the experimental groups. Furthermore, our research only studied the role of SFN in the early 72 hours post HS/R. The role of SFN in the late HS/R and the possibility of clinical application should be further confirmed. Finally, although designed to provide a clinically feasible setting, the complex injury pattern of our model is affected by many variables (such as drugs, infusions, diet, sample collection, etc.), and these variables may also affect the results.

## Conclusion

In conclusion, our present study demonstrated that *in vivo* SFN treatment down-regulates the proinflammatory cytokines expressed by Kupffer cells after isolated HS/R. This might serve as a potential therapeutic way to protect the liver from overwhelming local sterile inflammation and ischemia-reperfusion damage induced by isolated HS/R. SFN’s hepatic protective mechanism may be closely related with the Nrf2-ARE pathway activation of Kupffer cells and liver tissues. Further studies are required to elucidate the specific mechanisms and potential clinical applications of SFN.

## Data Availability Statement

The original contributions presented in the study are included in the article/**Supplementary Material**. Further inquiries can be directed to the corresponding author.

## Ethics Statement

The animal study was reviewed and approved by North Rhine-Westphalia Animal Welfare Committee.

## Author Contributions

WL, JG, FH and PL conceived the ideas and designed the study. WL and KQ performed laboratory work. WL, JG, AF, CW, TP and PK performed the data collection. All authors analyzed data and/or provided samples and reagents. WL and KQ wrote the paper with contributions from JG, AF, KH, FB, CW, TP, PK, FH and PL. All authors contributed to the article and approved the submitted version.

## Funding

This study was supported by the Braun Foundation (EMID: d2cd15afd6ed9f6d).

## Conflict of Interest

The authors declare that the research was conducted in the absence of any commercial or financial relationships that could be construed as a potential conflict of interest.

## Publisher’s Note

All claims expressed in this article are solely those of the authors and do not necessarily represent those of their affiliated organizations, or those of the publisher, the editors and the reviewers. Any product that may be evaluated in this article, or claim that may be made by its manufacturer, is not guaranteed or endorsed by the publisher.
