# Supplementary material for: Sulforaphane Exerts Beneficial Immunomodulatory Effects on Liver Tissue via a Nrf2 Pathway-Related Mechanism in a Murine Model of Hemorrhagic Shock and Resuscitation
Source: Front Immunol. 2022 Feb 10;13:822895. doi: 10.3389/fimmu.2022.822895 (PMC8866169; doi:10.3389/fimmu.2022.822895)
Supplement: Supplementary file 1 [file DataSheet_1.pdf]

frontiers

Supplementary Material

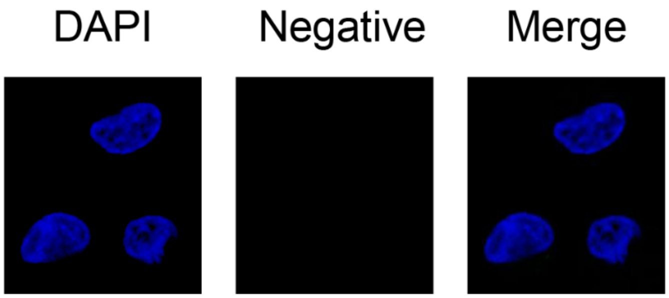

Supplementary Figure S1. Negative control of Kupffer cells' immunofluorescence.

Supplementary Table S1 Blood withdrawal

Group	HS/R (mL)						HS/R+SFN (mL)					
	**1**	**2**	**3**	**4**	**5**	**6**	**1**	**2**	**3**	**4**	**5**	**6**
**6h**	0.6	0.65	0.74	0.8	0.84	0.74	0.69	0.88	0.85	0.84	0.9	0.74
**24h**	0.65	0.84	0.74	0.79	0.71	0.89	0.65	0.71	0.63	0.81	0.71	0.76
**72h**	0.69	0.8	0.85	0.71	0.69	0.79	0.77	0.74	0.71	0.64	0.69	0.74
